# Supplementary material for: Human Serum Albumin-Based Nanoparticles for Targeted Intracellular Drug Delivery
Source: Int J Mol Sci. 2025 Aug 27;26(17):8297. doi: 10.3390/ijms26178297 (PMC12428110; doi:10.3390/ijms26178297)
Supplement: Supplementary file 1 [file ijms-26-08297-s001.zip › ijms-3728202-supplementary.pdf]

International Journal of Molecular Sciences

MDPI

Supplementary Information

Human Serum Albumin-Based Nanoparticles for Targeted Intracellular Drug Delivery

Claudia Gabriela Chilom [1], Sorina Iftimie [1], Adriana Elena Balan [1], Daniela Oprea [1,2] and Monica Enculescu [2], Teodor Adrian Enache [2,*]

[1] Faculty of Physics, University of Bucharest Magurele, 077125 Magurele, Ilfov, Romania; claudia.chilom@fizica.unibuc.ro (C.G.C.); sorina.iftimie@fizica.unibuc.ro (S.I.); adriana.balan@unibuc.ro (A.E.B.); daniela.oprea@infim.ro (D.O.)
[2] National Institute of Materials Physics, Str. Atomistilor, nr. 405A, 077125 Magurele, Ilfov, Romania; mdatcu@infim.ro
* Correspondence: adrian.enache@infim.ro

Evaluation of the stability of synthesized nanoparticles

Nanoparticles stability is a term that describes the preservation of a certain property of the nanostructure, such as aggregation, composition, crystallinity, shape, size, surface type (smooth or rough). For the investigation of these properties the HSA NPs and (HAS-FA):Ru NPs were stored at 4 ºC for up to 30 days. Their stability over time was analyzed by UV-Vis absorption spectroscopy, monitoring absorbance values at 280 nm, Figure S1. Through this method, the evaluation of the stability over time of the two types of NPs leded to the following conclusion: after about 20 days, the stability of HSA NPs was 54.9% and that of HSA-FA-Ru NPs was 68.8%.

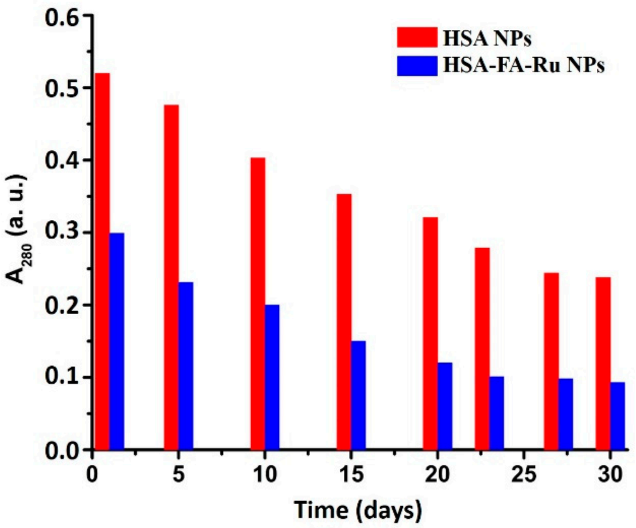

Figure S1. Time stability of HSA NPs and HSA-FA-Ru NPs obtained by desolvation method (desolvation agent ethanol, cross-linking agent glucose).

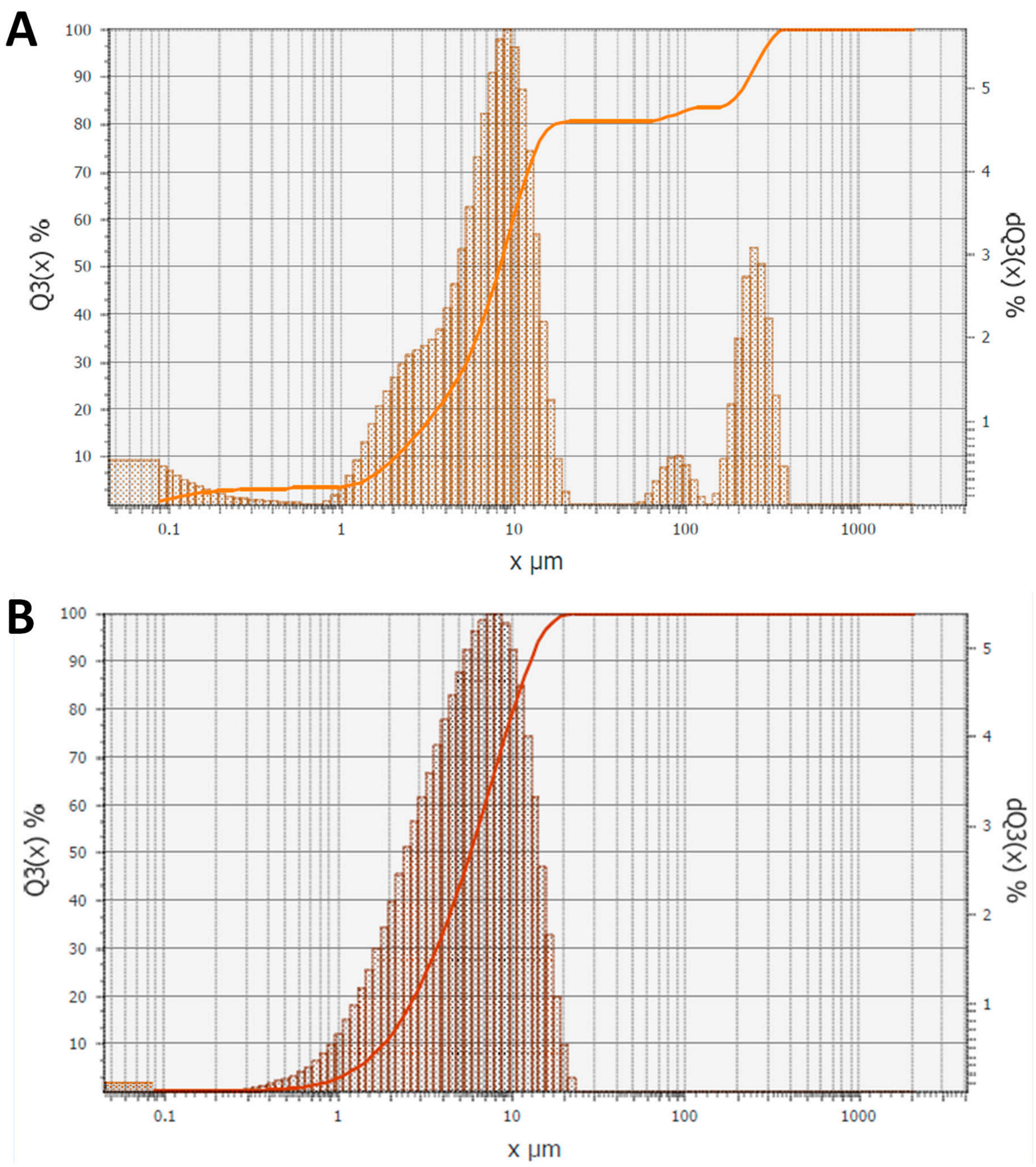

Figure S2. Size distribution of (A) HSA and (B) (HSA-FA):Ru suspensions obtained after 30 days incubation at 4 °C. Note that the x-axis (µm scale) is logarithmic.

Laser diffraction analysis of the 30 days incubated samples, on both HSA NPs and (HAS-FA):Ru NPs, revealed the presence of a high-abundance population with particle sizes around 6-7 µm in both nanoparticle systems. Importantly, precursor aggregates, corresponding to the initial nanoparticles, were still detected, with a size distribution below 100 nm. This observation suggests that, even after prolonged incubation, a fraction of the nanoparticles retains their original dimensions, while a significant proportion undergoes aggregation into larger structures, with differences that (HSA-FA):Ru presented aggregates around 10 µm compared with the sample without Ru, where the magnitude rich more than 500 µm.
